# OVERCOMING APATHY AMONG OLDER COMMUNITY DWELLERS IN RURAL NORTHERN BC: THE BENEFITS OF ENGAGING IN AN EBOOK CLUB

**DOI:** 10.1093/geroni/igae098.0921

**Published:** 2024-12-31

**Authors:** Aderonke Agboji, Shannon Freeman

**Affiliations:** University of Northern British Columbia, Prince George, British Columbia, Canada; University of Northern British Columbia, Prince George, British Columbia, Canada

## Abstract

The study aimed to investigate the impact of an eBook Club on the apathy and overall well-being of older adults dwelling in rural community settings. Employing a mixed-method approach, the research gathered data through surveys and interviews both before and after the program implementation. We recruited twenty-eight older adults, aged between 60 and 86 years, predominantly female (23 out of 28) from three communities in Northern British Columbia, Canada. Each participant was provided with a Kobo eReader loaded with books tailored to their interests, alongside a tutorial for using the device. Each eReader was linked to the public library, facilitating easy access to a broader range of books. The eBook Club convened weekly for sessions lasting 45 minutes to an hour, with groups capped at four members, over a span of 4 to 6 weeks. Initially, about half of the participants (10 out of 28) reported apathy. Remarkably, by the program’s conclusion, these individuals unanimously reported a decrease in apathetic feelings. Additional benefits noted by participants included enhanced mood, improved social connections, and sustained engagement with reading activities beyond the club meetings. We concluded that a relatively straightforward intervention like the eBook Club can significantly ameliorate symptoms of apathy and bolster the overall well-being of older individuals in rural community settings. While initial challenges with the Kobo eReader were reported, the collective support and encouragement within the group played a crucial role in overcoming these obstacles, underscoring the value of peer support in facilitating the adoption of new technologies.

